# Anatomical Factors in Children with Orbital Complications Due to Acute Rhinosinusitis

**Published:** 2019-09

**Authors:** Mustafa Çelik, Kamil-Hakan Kaya, Yakup Yegin, Burak Olgun, Fatma-Tülin Kayhan

**Affiliations:** 1 *Department of Otorhinolaryngology - Head and Neck Surgery, * *Kafkas University, Faculty of Medicine* *, Kars, Turkey.*; 2 *Department of Otorhinolaryngology - Head and Neck Surgery, Bakırköy Dr.Sadi Konuk Training and Research Hospital, Istanbul, Turkey.*

**Keywords:** Anatomy, Child, Sinusitis, Paranasal sinuses, Turbinates

## Abstract

**Introduction:**

The role of the anatomical variations and severity of acute rhinosinusitis (ARS) in the development of ARS complications is still an unknown issue. Regarding this, the present study evaluated the relationship between the severity of ARS and anatomical nasal variations in pediatric patients with ARS-related orbital complications.

**Materials and Methods:**

This study was conducted on 134 pediatric patients with orbital complications related to ARS. The data related to patients’ demographics, complication types, and involved side were collected. Nasal sides were also compared in terms of the Lund-Mackay score (LMS), osteomeatal complex (OMC) obstruction, Keros classification, presence of agger nasi cells (AGC), concha bullosa, Haller cells, Onodi cells, septal deviation, and lower turbinate hypertrophy.

**Results:**

The comparison of LMSs indicated a significant difference between the complicated and contralateral sides (8.37±2.44 vs. 5.62±2.71; P<0.0001). In addition, there was a significant difference between the complicated and contralateral sides in terms of the OMC scores (P<0.0001). The rates of lower turbinate hypertrophy and AGC on the complicated side were higher than those on the contralateral side (P=0.021 and P<0.00; respectively).

**Conclusion:**

As the results indicated, anatomical variability in adjacent structures affects the development of ARS-related orbital complications in pediatric patients.

## Introduction

Acute rhinosinusitis (ARS) is characterized by the inflammation of the mucous membranes in the nasal and paranasal sinuses (PNSs). The typical clinical manifestations of this infection are both mild and self-limiting (1). The ARS affects about 35 million people annually in the United States and leads to serious morbidity and impairments in the quality of life (1-3). 

The increased use of antibiotics, use of vaccines for bacterial rhinosinusitis (primarily caused by a pneumococcal infection), and easier access to radiological imaging methods, facilitating early diagnosis and treatment, have been accompanied by a reduction in the rate of ARS-related complications. However, these complications are still considered important health issues (4-8). Orbital complications related to ARS are commonly seen in pediatric patients (9). First, Hubert and later, Chandler categorized ARS complications based on their severity (6-9). Briefly, in this classification system, the ARS orbital complications were divided into five classes, namely inflammatory edema (class 1), orbital cellulitis (class 2), subperiosteal abscess (class 3), orbital abscess (class 4), and cavernous sinus thrombosis (class 5), respectively (9). It has been reported that 5-7% of patients with rhinosinusitis develop orbital complications (10-15). Orbital complications related to ARS are often seen in children; however, the prognosis of this disease in pediatric patients is reportedly better than that in adults (5). 

Several determinants, including age, gender, race, genetic factors, socioeconomic status, and environmental conditions, have been proposed to be responsible for the development of orbital complications related to ARS (10-21). The precise cause of ARS-related complications is unknown. The role of anatomical variations and severity of ARS in the development of complications is unclear yet. Furthermore, it is not clear why ARS complications are more common on one side. 

To the best of our knowledge, no study has investigated the associations of the side of orbital complications with the severity of ARS and anatomical variability in adjacent structures. With this background in mind, the present study evaluated the relationship of ARS severity and anatomical variations with orbital complications related to ARS. 

## Materials and Methods

The present retrospective study was performed on 134 pediatric patients (including 87 males and 47 females; mean age: 8.4±7.8 years; range: 7 months-18 years) with ARS-related orbital complications at our clinic from January 2011 to July 2016. Research approval was obtained from the Bakırköy Dr. Sadi Konuk Training and Research Hospital ethics commitee (ethical committee approval number: 2016-208/29.06.2016). The exclusion criteria were: 1) age of > 18 years, 2) immune deficiency, cystic fibrosis, nasal polyposis, and previous maxillofacial trauma history, 3) previous nasal or paranasal surgery, and 4) recurrent orbital complications related to ARS. The collected data included demographic information, complication types, and affected side.

All patients were evaluated according to the Chandler classification system (9). The patients with orbital complications related to ARS underwent the computed tomography (CT) of the PNS to evaluate disease recovery. All patients were informed about the side effects of the radiation, and CT was performed on patients providing informed consent. The PNS CT evaluation was performed using a multidetector CT scanner (Somatom Volume Zoom; Siemens Medical Solutions, Bonn, Germany). 

Contrast CT was not used, and the PNS CT imaging was performed using a Mediplus Dicom Wiever system (Mediplus Ltd., High Wycombe, UK). All PNS CT images were evaluated on a workstation (Leonardo; Siemens Medical Solutions) by two practiced otorhinolaryngologists. The severity of sinusitis was identified by using the Lund-Mackay score (LMS) based on the PNS CT images (16).

According to the LMS, each paranasal sinus was recorded as follows:

0: no opacity

1: partial lucency/opacity

2: complete opacity

A score of 0 and 2 was given for an obstructed and unobstructed osteomeatal complex (OMC), respectively. The maximum total score for each side was 12. Hypoplasia or agenesis of the PNS was noted on the CT scans. The depth of the olfactory fossa was identified by the extent of the lateral lamella of the cribriform plate. In 1962, Keros divided the depth of the olfactory fossa into three types as follows (3): 

Type I: a depth of 1-3 mm

Type II: a depth of 4-7 mm 

Type III: a depth of 8-16 mm 

The sides were also compared in terms of the Keros classification, presence of agger nasi cells (AGC), concha bullosa, Haller, Onodi or frontal recess cells, septal deviation, and lower turbinate hypertrophy.

**Statistical analysis**

The Number Cruncher Statistical System 2007 Statistical Software (NCCS LLC, Kaysville, UT, USA) was used for statistical analysis. The data were analyzed using descriptive statistical methods (mean and standard deviation), one-way analysis of variance (for group comparison), Tukey’s multiple comparison test (for subgroup comparison), independent t-test (for comparing two sides), and Chi-square tests (for comparing qualitative data). A p-value less than 0.05 was considered statistically significant.

## Results

The complications observed in the participants included inflammatory edema (n=112, 83.6%), orbital cellulitis (n=6, 4.4%), subperiosteal abscess (n=15, 11.3%), and orbital abscess (n=1, 0.7%). Cavernous sinus thrombosis was not detected in any of the patients. Complications were found on both sides equally. The OMC opacification was found in 124(92.5%) and 63 (47.0%) complicated and contralateral sides, respectively. Evaluation of LMS according to the sinuses and OMC indicated a significant difference between complicated and uncomplicated sides in all sinuses (all P<0.05; Table. 1). 

**Table 1:** Comparison of sinuses in terms of Lund Mackay scores

		**Complicated side**		**Uncomplicated side**		a **P**
		**n**	**%**	**n**	**%**	
Maxillary sinus	Complete lucency	4	2.9%	19	14.1%	0.0001
	Partial lucency	60	44.7%	72	53.7%	
	Complete opacity	70	52.4%	43	32.2%	
Anterior ethmoid sinus	Complete lucency	0	0.0%	62	46.3%	0.0001
	Partial lucency	54	40.3%	48	35.8%	
	Complete opacity	80	59.7%	24	17.9%	
Posterior ethmoid sinus	Complete lucency	8	5.95%	42	31.3%	0.0001
	Partial lucency	47	35.1%	66	49.2%	
	Complete opacity	79	58.95%	26	19.5%	
Frontal sinus	Complete lucency	10	7.5%	58	43.3%	0.0001
	Partial lucency	52	38.8%	44	32.8%	
	Complete opacity	72	53.7%	32	23.9%	
Sphenoid sinus	Complete lucency	21	15.7%	36	26.9%	0,0001
	Partial lucency	69	51.5%	77	57.5%	
	Complete opacity	44	32.8%	21	15.6%	

a **P:** One-way analysis of variance (ANOVA) test

The comparison of LMSs indicated a significant difference between the complicated and contralateral sides (8.37±2.44 vs. 5.62±2.71, respectively; P<0.0001). In addition, septal deviation was found in 43 (32.1%) complicated sides and 39 (29.1%) contralateral sides. The results revealed no significant difference between the complicated and contralateral sides regarding the presence of septal deviation (P=0.344 and P>0.05, respectively).

The AGC was seen in 68 (50.7%) and 44 (32.8%) complicated and contralateral sides, respectively. The frequency of AGC was significantly higher in the complicated sides than in the contralateral sides (P=0.0001). Furthermore, concha bullosa was found in 42 (31.3%) and 38 (28.4%) complicated and contralateral sides, respectively. However, this difference was not statistically significant (P=0.388). Lower turbinate hypertrophy was detected in 64 (47.8%) and 46 (34.3%) complicated and contralateral sides, respectively. In this regard, the rate of lower turbinate hypertrophy was significantly higher in the complicated sides than in the contralateral sides (P=0.021). In terms of the Keros type distributions, types 1, 2, and 3 of this condition were observed in 63 (47.0%), 59 (44.0%), and 12 (9.0%) complicated sides, respectively. In addition, 45 (33.6%), 58 (43.3%), and 31 (23.1%) cases of types 1, 2, and 3 were observed in the contralateral sides, respectively. The results revealed a significant difference between the two sides in terms of the Keros type distribution (P=0.001*; *Table.2).

**Table 2:** Comparison of the complicated and contralateral sides in terms of the Keros classification system

		**Complicated side**				**Uncomplicated side**			**P**
		**n**		**%**		**N**		**%**	
Keros classification	Type 1	63		47,0%		45		33,6%	0.000 N
	Type 2	59		44,0%		58		43,3%	
	Type 3	12		9,0%		31		23,1%	

N McNemar’s test

The results revealed no significant difference between the two sides regarding the presence of Haller or Onodi cells(P=0.344 and P=0.109, respectively).The results are shown in Table 3. 

**Table 3:** Comparison of radiological findings in the complicated and contralateral sides

		**Complicated side**				**Contralateral side**				**P**	
		N	%		n	%					
Osteomeatal complex opacification	(-)	10	7.5%		71	53.0%			<0.0001	^N^	
	(+)	124	92.5%		63	47.0%					
Septal deviation	(-)	91	67.9%		95	70.9%			0.344	^N^	
	(+)	43	32.1%		39	29.1%					
Agger nasi cell	(-)	66	49.3%		90	67.2%			<0.0001	^N^	
	(+)	68	50.7%		44	32.8%					
Concha bullosa	(-)	92	68.7%		96	71.6%			0.388		
	(+)	42	31.3%		38	28.4%					
Lower turbinate hypertrophy	(-)	70	52.2%		88	65.7%			0.021		
	(+)	64		47.8%		46		34.3%			
Haller cell	(-)	124		92.5%		128		95.5%		0.344	^N^
	(+)	10		7.5%		6		4.5%			
Onodi cell	(-)	125		93.3%		131		97.8%		0.109	^N^
	(+)	9		6,7%		3		2,2%			
Lund-Mackay scores	8,037	±	2,44			5,62	±	2,71		<0.0001	

N McNemar’s test;

M Mann-Whitney U test

## Discussion

The lack of data regarding the factors accounting for the development of ARS-related orbital complications obscures the management of several contentious topics. Regarding this, the determination of such factors is an important issue in the field of otolaryngology. Adequate knowledge about the preventable causes of ARS-related orbital complications will reduce the resource-intensive and significant economic burdens associated with these complications; however, many questions in this domain have remained unanswered. Schollin Ask et al. reported the orbital complications as the most common cause of hospitalization in pediatric patients with ARS (12). They also demonstrated that the boys under the age of 2 years were the most common hospitalized pediatric patients.

The classification of ARS complications was first performed by Hubert in 1937 (6-8). Therefore, a new suitable classification system was required. Smith and Spencer in 1948 and Chandler et al. in 1970 published new classifications (9,19). The Chandler system classifies the severity of complications rather than the different stages of disease. Despite the widespread use of Chandler system, a new classification system published by Mortimore and Wormald in 1977 (called the “Groote Schuur hospital classification system”) increased the confusion of the terms related to the identification of different forms of orbital complications (20). Finally, Velasco e Cruz et al. divided the orbital complications into orbital cellulitis, subperiosteal abscess, and orbital abscess (21). In the present study, the Chandler classification system was applied. 

The sphenoid sinuses and posterior ethmoids clear out through the superior turbinate, while all other sinuses drain into the OMC, which is bordered by the uncinate process anteriorly and inferiorly, the middle turbinate medially, the lamina papyracea laterally, and the ethmoid bulla posteriorly. The OMC drains through the infundibulum located inferiorly and bordered by the uncinate process anteroinferiorly (11-18). Developmental anomalies in the anatomy of the sinuses and drainage pathways may predispose adults and children to acute and chronic sinusitis at similar frequencies (19-26). Few studies have investigated the relationships between ARS-related orbital complications and anatomical variability in the sinonasal region. Shing et al. suggested that orbital complications related to ARS in children are mostly seen on one side and may depend on dissymmetries in the anatomical structure of the lamina papyracea (27). These asymmetries are distributed asymmetrically on the lamina papyracea and may facilitate the development of orbital complications. In the present study, asymmetries in the anatomical structure of the lamina papyracea could not be assessed due to the difficulty of the task caused by opacity in the adjacent anatomical structures. 

Thorp et al. compared the infundibular width and length, as well as uncinate angle between 24 complicated sides and 196 healthy children (28). According to their results in complicated sinusitis, mucosal OMC pathologies seem more than bony pathologies. Although they did not measure the contralateral side, they noted that the dimensions on the unaffected side were not markedly different from those on the affected side. 

The present study is the first anatomical CT study comparing the complicated and contralateral sides in children with orbital complications related to ARS in terms of PNS anatomical variations. As evaluated by the Lund-Mackay staging, the two sides differed significantly in terms of mucosal disease. The LMS was higher in the complicated sides than in the contralateral sides. In addition, the rates of AGC and the rates of lower turbinate hypertrophy were higher in the complicated side than in the contralateral sides. Furthermore, the OMC obstruction was higher in the complicated sides than in the contralateral sides. 

It was concluded that the higher severity of ARS may facilitate the development of ARS-related complications. In addition, the presence of AGC and lower turbinate hypertrophy may cause anatomical variability in the OMC region. This condition may facilitate higher rates of OMC obstruction and complication development. To the best of our knowledge, there are no studies regarding the effects of sinonasal anatomical variability and severity of ARS on the development of orbital complications related to ARS in children. 

Clinicians should be aware of the anatomical variability of the sinonasal region in pediatric patients presenting with orbital complications due to ARS. Serious orbital complications can be prevented with early medical interventions. A delay in the determination of the potential risk factors for orbital complications, including anatomical variability, may result in the development of more serious complications. Better conceptualization of the reasons for orbital complications would be beneficial. Surgical treatment must be considered in cases with anatomical variations. 

Although we obtained interesting results, our study has some limitations. This study did not focus on the dimensions (e.g., thickness, length, and volume) of the anatomical structures or variations. Other limitations of the study were the retrospective collection of data and the lack of randomization. There is no prospective study investigating this domain; therefore, the sample size of the study was small. Diagnostic issues for the confirmation of an extraneous infection in pediatric patients with ARS remain unclear. Nasal endoscopic evaluation might be uneasy. Furthermore, CT does not represent an absolute means of differentiation between viral and bacterial parasinusal infections. 

## Conclusion

Anatomical variability in adjacent structures affects the development of orbital complications related to ARS in pediatric patients. As the findings indicated, the LMS was higher on the complicated side, compared with that on the contralateral side. However, additional studies are obligatory to confirm these initial findings.
